# Multi-Strategy Honey Badger Algorithm for Global Optimization

**DOI:** 10.3390/biomimetics10090581

**Published:** 2025-09-02

**Authors:** Delong Guo, Huajuan Huang

**Affiliations:** 1 School of Mathematics and Statistics, Qiannan Normal University for Nationalities, Duyun 558000, China; 2 Guangxi Key Laboratory of Hybrid Computation and IC Design Analysis, Guangxi University for Nationalities, Nanning 530006, China; 3 College of Artificial Intelligence, Guangxi University for Nationalities, Nanning 530006, China; hhj-025@163.com

**Keywords:** honey badger algorithm, chaos mapping, random perturbation strategy, elite tangent search, differential mutation, engineering application issues

## Abstract

The Honey Badger Algorithm (HBA) is a recently proposed metaheuristic optimization algorithm inspired by the foraging behavior of honey badgers. The search mechanism of this algorithm is divided into two phases: a mining phase and a honey-seeking phase, effectively emulating the processes of exploration and exploitation within the search space. Despite its innovative approach, the Honey Badger Algorithm (HBA) faces challenges such as slow convergence rates, an imbalanced trade-off between exploration and exploitation, and a tendency to become trapped in local optima. To address these issues, we propose an enhanced version of the Honey Badger Algorithm (HBA), namely the Multi-Strategy Honey Badger Algorithm (MSHBA), which incorporates a Cubic Chaotic Mapping mechanism for population initialization. This integration aims to enhance the uniformity and diversity of the initial population distribution. In the mining and honey-seeking stages, the position of the honey badger is updated based on the best fitness value within the population. This strategy may lead to premature convergence due to population aggregation around the fittest individual. To counteract this tendency and enhance the algorithm’s global optimization capability, we introduce a random search strategy. Furthermore, an elite tangential search and a differential mutation strategy are employed after three iterations without detecting a new best value in the population, thereby enhancing the algorithm’s efficacy. A comprehensive performance evaluation, conducted across a suite of established benchmark functions, reveals that the MSHBA excels in 26 out of 29 IEEE CEC 2017 benchmarks. Subsequent statistical analysis corroborates the superior performance of the MSHBA. Moreover, the MSHBA has been successfully applied to four engineering design problems, highlighting its capability for addressing constrained engineering design challenges and outperforming other optimization algorithms in this domain.

## 1. Introduction

Optimization refers to the process of finding the best solution for a given system from all possible values to maximize or minimize the output. Over the past few decades, as the complexity of problems has increased, the demand for new optimization techniques has become more pressing [1,2]. In the past, traditional mathematical techniques used to solve optimization problems were mostly deterministic, but a major issue was their tendency to become trapped in local optima. This has resulted in low efficiency when using these techniques to solve practical optimization problems over the past two decades [3,4], thereby increasing interest in stochastic optimization techniques. In general, most real-world optimization problems—such as those in engineering [5], wireless sensor networks [6], image processing [7], feature selection [8,9], tuning machine learning parameters [10], and bioinformatics [11]—are highly nonlinear and non-convex due to inherent complex constraints and numerous design variables. Therefore, solving these types of optimization problems is highly complex due to the presence of numerous inherent local minima. Additionally, there is no guarantee of finding a global optimal solution.

Optimization problem-solving algorithms are classified into five main categories based on heuristic creation principles. Human-based optimization algorithms are designed based on human brain thinking, systems, organs, and social evolution. An example is the well-known Neural Network Algorithm (NNA) [12], which solves problems based on the message transmission in neural networks of the human brain. The Harmony Search (HS) algorithm [13,14] simulates the process by which musicians achieve a harmonious state by iteratively adjusting pitches through memory recall.

Algorithms that mimic natural evolution are classified as evolutionary optimization algorithms. The Genetic Algorithm (GA) [15] is the most classic model that simulates evolution, where chromosomes form offspring through a series of stages in cycles and produce more adaptive individuals through selection and reproduction mechanisms. Additionally, the differential evolution (DE) algorithm [16,17], the Imperialist Competitive Algorithm (ICA) [18], and the Mimetic Algorithm (MA) [19] are also based on evolutionary mechanisms.

Population-based optimization algorithms simulate the behaviors of biological populations, including reproduction, predation, and migration. In these algorithms, individuals in the population are treated as massless particles searching for the best position. The Ant Colony Optimization (ACO) algorithm [20,21] utilizes the concept of ants finding the shortest path from the nest to food sources. The Particle Swarm Optimization (PSO) algorithm [22] is derived from the foraging behavior of birds and is widely recognized as a swarm intelligence algorithm. The Moth Flame Optimization (MFO) algorithm [23] is a mathematical model that simulates the unique navigation behavior of moths, which spiral towards a light source until they reach the “flame.” Other swarm intelligence algorithms include the Grey Wolf Optimization (GWO) algorithm [24], the Moth-Flame Optimization (MFO) algorithm [25], the Artificial Hummingbird Algorithm (AHA) [26], the Stinky Pete Optimization (DMO) algorithm [27], the Chimpanzee Optimization Algorithm (CHOA) [28,29], the Raccoon Optimization Algorithm (COA) [30], the Beetle Optimization Algorithm (DBO) [31], Harris Hawk Optimization (HHO) [32], and the Osprey Optimization Algorithm (OOA) [33].

Plant growth-based optimization algorithms. The inspiration for these algorithms comes from plant characteristics such as photosynthesis, flower pollination, and seed dispersal. The Dandelion Optimization (DO) algorithm [34] is inspired by the processes of rising, falling, and landing of dandelion seeds in various wind directions. Algorithms that mimic the aggressive invasion of weeds, their search for suitable living spaces, and their utilization of natural resources for rapid growth and reproduction are known as Invasive Weed Optimization (IWO) [35].

Physics-based optimization algorithms are developed based on natural physical phenomena and laws. The Gravity Search Algorithm (GSA) [36] originates from the concept of gravity, and it possesses powerful global search capabilities and fast convergence speed. The Artificial Raindrop Optimization Algorithm (ARA) [37] is designed based on the processes of raindrop formation, landing, collision, aggregation, and evaporation into water vapor.

In particular, due to their excellent performance, many algorithms have been applied to a wide range of practical engineering problems, such as feature selection [38,39,40], image segmentation [41,42], signal processing [43], hydraulic facility construction [44], walking robot path planning [45,46], job shop scheduling [47], and pipeline and wiring optimization in industrial and agricultural production [48]. Unlike gradient-based optimization algorithms, metaheuristic algorithms rely on probabilistic searches rather than gradient-based methods. In the absence of centralized control constraints, the failure of individual agents will not affect the overall problem-solving process, ensuring a more stable search process. Typically, as a first step, it is necessary to appropriately set the basic parameters of the algorithm and generate an initial population of random solutions. Next, the search mechanism of the algorithm is employed to locate the optimal value until a stopping criterion is met or the optimal value is identified [49]. However, it is evident that each algorithm possesses distinct advantages and disadvantages, and its performance may vary depending on the specific problem being addressed. The “No Free Lunch” (NFL) theorem [50] posits that while an algorithm may effectively solve certain optimization problems, there is no universal guarantee that it can successfully address other optimization problems. Therefore, when confronted with several specific problems, it is reasonable to propose multiple strategies to enhance the efficiency of the algorithm.

In order to identify more effective problem-solving approaches, numerous researchers have endeavored to develop new algorithms and enhance existing methods. Research on metaheuristic optimization algorithms has led to the development of effective search strategies for achieving global optimality. Due to the exponential growth of the search space in real-life optimization problems, which often exhibit multimodality, traditional optimization methods frequently yield suboptimal solutions. Over the past few decades, the development of numerous new metaheuristic algorithms [51] has demonstrated robust performance across a broader spectrum of complex problems.

The Honey Badger Algorithm (HBA) is a novel metaheuristic algorithm proposed by Fatma A. Hashim et al. in 2022, inspired by the foraging behavior of honey badgers in nature. The algorithm searches for the optimal solution to problems by simulating the dynamic foraging and digging behaviors of honey badgers. The algorithm is known for its strong search ability and fast convergence speed compared to other algorithms, but it also has the disadvantage of slower search performance in the late stages and a tendency to become trapped in local optimal solutions.

The proposed Multi-Strategy Honey Badger Algorithm (MSHBA) is introduced to address the aforementioned issues. Due to the random generation of initial populations in the basic Honey Badger search algorithm, it cannot guarantee the uniform distribution of individuals within the search space, thereby affecting the algorithm’s search efficiency and optimization performance. The Cubic chaos mapping mechanism is incorporated into the initialization process of the improved Honey Badger algorithm to enhance the traversal capability of the initial population. During the digging and honey-seeking phases of the Honey Badger Algorithm, the positions of the agents are updated based on the best value within the population. This approach can lead to premature convergence due to population clustering around the optimal individual. To enhance the global optimization capability of the Honey Badger Algorithm, a random search strategy is introduced. When the population identifies the same best value for three consecutive iterations, both the elite tangent search and the differential mutation strategy are executed. Finally, the MSHBA algorithm is tested and validated on the CEC 2017 benchmark suite, demonstrating improved convergence speed and accuracy, as well as high efficiency in solving engineering problems.

## 2. Fundamentals of Honey Badger Algorithm

In the standard Honey Badger Optimization Algorithm, the optimal solution to the optimization problem is obtained by updating the prey odor intensity factor and employing two distinct foraging strategies of honey badgers: the “digging phase” and the “honey phase,” each characterized by unique search trajectories.

During the initialization stage, the population size of honey badgers is N, and the position of each individual honey badger is determined by the following equation:

(1) $$x_{i}=lb_{i}+r_{1}\times (ub_{i}-lb_{i})$$

where r is a random number between 0 and 1, $x_{i}$ is the position of the i-th honey badger referring to a candidate solution, while $lb_{i}$ and $ub_{i}$ are the lower and upper bounds of the search space, respectively.

Defining Intensity (I): Intensity is related to the concentration of prey and the distance of individual honey badgers. $I_{i}$ represents the intensity of the scent. If the odor concentration is high, the honey badger searches for prey more rapidly; conversely, if the concentration is low, the search is slower. Therefore, the intensity of the scent is directly proportional to the concentration of the prey and inversely proportional to the square of the distance from the honey badger. The specific definition is given by the following equation:

(2) $$\begin{array}{l} I_{i}=r_{2}\times \frac{S}{4\pi d_{i}^{2}}\\ S={\left(x_{i}-x_{i+1}\right)}^{2}\\ d_{i}=x_{prey}-x_{i} \end{array}$$

where $r_{2}$ is a randomly generated number ranging from 0 to 1, S is the prey concentration, and $d_{i}$ indicates the distance between the prey and the first badger.

Update the density factor. The density factor α controls the time-varying randomization to ensure a smooth transition from exploration to exploitation; as the number of iterations increases, the intensity factor decreases, thereby reducing randomization over time, as given by the equation below:

(3) $$\alpha =C\times \exp(\frac{-t}{t_{\max}})$$

where $t_{\max}$ is the maximum number of iterations, and C is a constant ≥1 (default = 2).

Digging phase

At this stage, the honey badger automatically locates beehives by scent and destroys them to obtain food. Its path follows the shape of a heart, and its location is updated as follows:

(4) $$x_{new}=x_{prey}+F\times \beta \times I\times x_{prey}+F\times r_{3}\times \alpha \times d_{i}\times \left|\cos(2\pi r_{4})\times \left[1-\cos(2\pi r_{5})\right]\right|$$

(5) $$F=\left\{\begin{array}{l} 1,r_{6}\leq 0.5\\ -1,r_{6}>0.5 \end{array}\right.$$

where $x_{prey}$ is the position of the prey, which is the best position found so far—in other words, the global best position. β≥1 (default = 6) is ability of the honey badger to obtain food. $d_{i}$ is the distance between the prey and the ith honey badger, see Equation (2). $r_{3}$, $r_{4}$, and $r_{5}$ are three different random numbers between 0 and 1. F works as the flag that alters search direction; it is determined using Equation (5), where $r_{6}$ is a randomly generated number ranging from 0 to 1.

Honey phase

At this stage, the honey badger follows the honey guide bird to the beehive to find honey. This process can be described by the following formula:

(6) $$x_{new}=x_{prey}+F\times r_{7}\times \alpha \times d_{i}$$

Here, $x_{new}$ refers to the new position of the honey badger, whereas $x_{prey}$ denotes the location of the prey. The parameters F and α are determined using Equations (3) and (5), respectively. From Equation (6), it can be observed that the honey badger performs a search in the vicinity of the best $x_{prey}$ location found so far, based on distance information $d_{i}$. At this stage, the search behavior is influenced by a time-varying factor t. Moreover, the honey badger may encounter disturbances F, where r is a random number between 0 and 1.

## 3. Multi-Strategy Honey Badger Algorithm

Given the shortcomings of the standard HBA algorithm, such as its tendency to easily fall into local optima and its slow convergence speed in the later stages, this paper introduces a cubic chaotic mapping mechanism during the population initialization phase and incorporates a random search strategy into the position update process of the honey badgers. When the global best solution obtained by the honey badger population remains unchanged for three consecutive iterations, the elite tangent search and differential mutation strategies are introduced.

### 3.1. Cubic Chaotic Mapping

Since the initial population of the basic Honey Badger Algorithm is generated randomly, the uniform distribution of individuals in the search space cannot be guaranteed, which may negatively impact the convergence speed and optimization performance of the algorithm. To improve the initialization process of the Honey Badger Algorithm, cubic mapping [52] was introduced to enhance the diversity and coverage of the initial population, as shown below:

(7) $$x_{i}=lb_{i}+(ub_{i}-lb_{i})\times z_{i},i=1,2,\cdots ,n$$

(8) $$z_{i+1}=\rho \times z_{i}(1-z_{i}^{2})$$

where $x_{i}$ denotes the position of the i-th honey badger; $lb_{i}$ and $ub_{i}$ represent the lower and upper bounds of the i-th variable, respectively; $z_{i}$ is a constant; and ρ is the cubic chaotic sequence, typically set to a constant value of 3. This method generates individuals in the initial population to ensure a uniform and diverse distribution across the search space.

### 3.2. Introduction of Random Value Perturbation Strategy for Honey Badger Algorithm

In the Honey Badger Algorithm, the position of each honey badger is updated based on the global best solution $x_{prey}$, which may easily result in premature convergence as the population tends to cluster around the current best individual. To enhance the global optimization capability of the Honey Badger Algorithm, a random search strategy [53] is incorporated, and the position update mechanism is adaptively determined based on the value of a control coefficient A. When the specified condition is $\left|A\right|\geq 1$, a perturbation-based search strategy is applied to randomly selected individuals; otherwise, the position of the honey badger is updated based on the current global best solution $x_{prey}$. The expression is given by

(9) A=2⋅m⋅cos(r)−m 

In this expression, $m=2(1-\frac{t}{t_{\max}})$, r denotes a random number drawn from the interval (0,1). Its value decreases linearly from 2 to 0, where r denotes a random number drawn from the interval (0,1). It can be seen from the above two expressions that the value of m generally shows a linear decreasing trend. In the early stages of iteration, the algorithm repeatedly applies random search strategies to prevent premature convergence due to population clustering, enhance the exploration capability of honey badgers across the search space, and improve global search performance.

The mathematical expressions for the digging phase and the honey phase of the Honey Badger Algorithm at the current stage are given as follows:

(10) $$x_{new}=x_{rand}+F\times \beta \times I_{i}\times rand+F\times r_{3}\times \alpha \times d_{i}\times \left|\cos(2\pi r_{i})\times [1-\cos(2\pi r_{5})\right|\;$$

(11) $$x_{new}=x_{rand}+F\times r_{7}\times \alpha \times d_{i}\;$$

where $d_{i}=x_{rand}-x_{i}$.

### 3.3. Population Remains the Best Value Unchanged After Three Iterations, Elite Tangent Search and Differential Variation Strategy Are Executed

During each iteration, the population is divided into two subpopulations: the first half, consisting of individuals with lower fitness values, is designated as the elite subpopulation, while the second half serves as the exploration subpopulation. The elite subpopulation then executes the elite migration strategy.

#### 3.3.1. Migration Strategy of Elite Subpopulation

When an individual approaches the current optimal solution, it explores the surrounding region—a behavior referred to as local search—which enhances convergence speed and solution accuracy. Since the fitness of the elite subpopulation is close to the current global best solution, enabling it to perform local search can improve the convergence rate and solution precision. The migration strategy of the elite subpopulation [54] involves updating the position by incorporating the Tangent Search Algorithm [55] (TSA). The mathematical formulation of the elite tangent search strategy is given as follows:

(12) $$x=x_{prey}+step\times \tan(\theta )\times (rand\times x_{prey}-x),if\;\;\;\;\;x=opts\;$$

(13) $$x=x_{prey}+step\times \tan(\theta )\times (x_{prey}-x),if\;\;\;\;\;x\neq opts\;$$

Due to the perturbation applied to elite individuals, the original position x is transformed to a new position $x_{prey}$.

#### 3.3.2. Exploration of Subpopulation Evolution Strategies

In the Honey Badger Algorithm, the position updating mechanism generates new individuals in the vicinity of the current individual x and the current global best individual $x_{prey}$. In other words, other individuals in the population are guided toward the global best solution. However, if this solution is a local optimum, continued iterations may cause the honey badger individuals to converge around it, resulting in reduced population diversity and increasing the risk of premature convergence. To address these issues, a differential mutation strategy is adopted. Inspired by the mutation strategy in differential evolution, the current individual, the global best individual, and randomly selected individuals from the population are used to perform differentiation in order to generate new individuals. This process is described by the following equation:

(14) $$X(t+1)=X_{rand1}(t)+F_{0}\times (X_{rand2}(t)-X_{rand3}(t))\;$$

where $F_{0}=0.4$ is the scaling factor for differential evolution; *t* denotes the current iteration number; and $X_{rand1},X_{rand2},X_{rand3}$ represent three randomly selected honey badger individuals.

**Figure 1:**
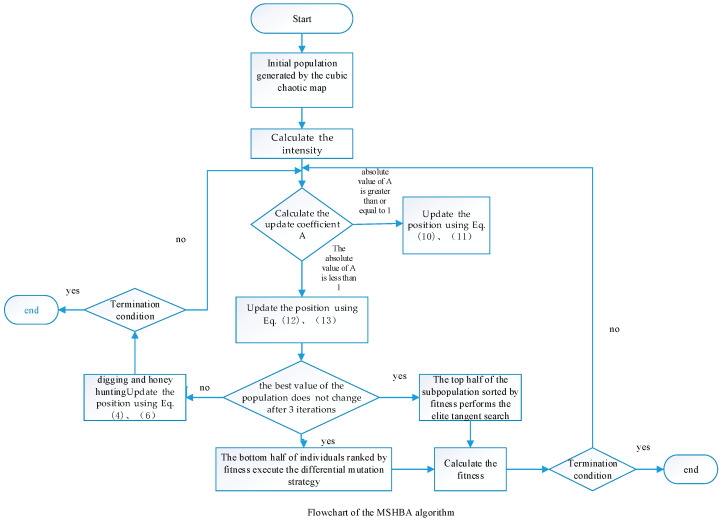
Flowchart of the MSHBA algorithm.

The flowchart of the MSHBA algorithm is shown in Figure 1. Algorithm 1 presents the pseudocode of MSHBA.
**Algorithm 1** Pseudo Code of MSHBASet parameters $T_{\max}$, N,β,C. Initialize population using Equations (7) and (8) **while**
$t\leq T_{\max}$ **do** Update the decreasing factor α using (3). **for** *i* = 1 to *N*
**do**  Calculate the intensity $I_{x}$ using Equation (2).  Calculate A using Equation (9).  **if**
$\left|A\right|\geq 1$   Update the position $x_{new}$ using Equations (10) and (11).  **else**   Update the position $x_{new}$ using Equations (5) and (6).  **end.**  If the best value of the population does not change after 3 iterations    The first half of the subpopulation performs the elite tangent search strategy.   Update the position $x_{new}$ using Equations (12) and (13).   The second half of the subpopulation performs the differential mutation strategy.   Update the position $x_{new}$ using Equation (14).    else   digging and honey hunting   Update the position using Equations (4) and (6)  **end**  **If** stop criteria satisfied.  Output the optimal solution.  else  return Calculate A using Equation (9).  end

## 4. Experimental Results and Discussion

The experiments were conducted on the Windows 10 operating system. All algorithms were implemented using MATLAB R2023b. The performance of the IHBA was compared with that of recently developed metaheuristics to evaluate its effectiveness in global optimization. The numerical efficiency of MSHBA was evaluated using 29 benchmark functions from CEC 2017 and four engineering design problems. To validate the performance of MSHBA, the results were compared with four state-of-the-art optimization algorithms: COA [30], DBO [31], HHO [32], and OOA [33]. Among the selected competitive algorithms, COA, DBO, HHO, and OOA are swarm intelligence algorithms widely recognized in the metaheuristic literature. On the other hand, COA, DBO, HHO, and OOA are relatively recent algorithms that have demonstrated promising performance in addressing the optimization problems considered in this study. These methods were selected to include both well-established and recently proposed algorithms, ensuring a fair comparison to demonstrate the overall effectiveness of the proposed approach. For a fair comparison, a maximum of 500 iterations was set for each optimization problem.

### 4.1. Parameter Settings

Apart from the algorithm-specific parameter settings and the dimensions of the test functions listed in Table 1 and Table 2, the general settings common to all selected algorithms include a population size of 30 (N=30), a maximum of 500 iterations ($T_{\max}=500$), and 30 independent runs for each optimization problem.

### 4.2. Benchmark Testing Functions

The performance of the Improved Honey Badger Optimization Algorithm is evaluated using the CEC2017 benchmark functions, which are categorized into unimodal functions, basic multimodal functions, hybrid functions, and composite functions, as presented in the Table 1 below.

Function F2 has been excluded from the benchmark set, as it exhibits unstable behavior, particularly in high-dimensional cases, and shows significant variations in performance when the same algorithm is implemented in MATLAB.

### 4.3. Comparison of MSHBA Algorithm with Other Algorithms

Table 3 demonstrates that the MSHBA algorithm achieves superior performance in terms of minimum, worst-case, median, average, and standard deviation (std) values on the unimodal functions F1 and F3, compared to the Raccoon Optimization Algorithm (COA), Dung Beetle Optimization (DBO), Harris Hawk Optimization (HHO), Osprey Optimization Algorithm (OOA), and the original Honey Badger Algorithm (HBA). These results indicate a significant improvement in the performance of the enhanced Honey Badger Algorithm (MSHBA).

Table 4 shows that the MSHBA algorithm outperforms the Raccoon Optimization Algorithm (COA), Dung Beetle Optimization (DBO), Harris Hawk Optimization (HHO), Osprey Optimization Algorithm (OOA), and the original Honey Badger Algorithm (HBA) when tested on a set of multimodal functions (F4–F10), in terms of minimum, worst-case, median, average, and standard deviation (std) values.

From Table 5, it can be seen that the MSHBA algorithm exhibits superior performance min, worst, median, average, and std on the hybrid function set F11–F20 compared to other algorithms such as Raccoon Optimization Algorithm (COA), Beetle Optimization Algorithm (DBO), Harris Hawk Optimization (HHO), Ospry Optimization Algorithm (OOA), Honey Badger Algorithm (HBA).

Table 6 shows that the MSHBA algorithm demonstrates superior performance in terms of minimum, worst-case, median, average, and standard deviation (std) values on the hybrid functions F21, F23, F24, F26, and F30, compared to other algorithms such as the Raccoon Optimization Algorithm (COA), Dung Beetle Optimization (DBO), Harris Hawk Optimization (HHO), Osprey Optimization Algorithm (OOA), and the original Honey Badger Algorithm (HBA). However, its performance in terms of variance is slightly inferior to that of COA on function F22, and to that of HBA on functions F25 and F29. Additionally, the mean and variance performances are slightly weaker than those of HBA on functions F27 and F28.

### 4.4. MSHBA and Other Algorithms’ Rank-Sum Test

Table 7 shows that the Multi-Strategy Honey Badger Optimization Algorithm (MSHBA) demonstrates significantly superior performance compared to the other four algorithms (COA, DBO, OOA, and HHO) on test functions F1 and F3–F30, according to the Wilcoxon rank-sum test, as the obtained *p*-values are significantly lower than the given significance level. Overall, the improved algorithm outperforms the other four algorithms. However, on test functions F1, F12, F15, F18, F19, F20, F22, F25, F27, F28, and F29, the performance of the improved Honey Badger Algorithm is comparable to that of the original Honey Badger Algorithm. In general, the performance of the Honey Badger Algorithm has been significantly enhanced by introducing hybrid strategy operators.

### 4.5. MSHBA and Other Algorithms of Boxplot

**Figure 2:**
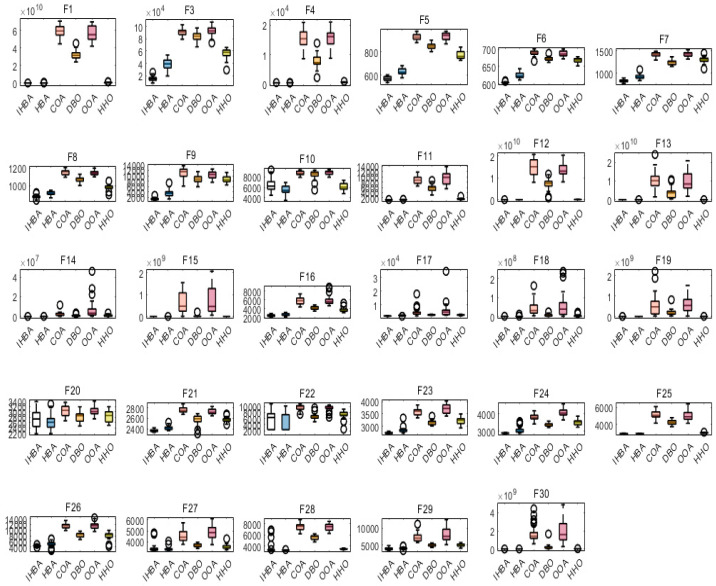
Boxplot Comparison of MSHBA with Other Algorithms.

Figure 2 Boxplot Comparison of MSHBA with Other Algorithms outperforms the Raccoon Optimization Algorithm (COA), Dung Beetle Optimization (DBO), Harris Hawk Optimization (HHO), Osprey Optimization Algorithm (OOA), and the original Honey Badger Algorithm (HBA) on the CEC2017 test functions F3, F5–F9, F12, F13, F15–F19, F21, F23, F24, F26, F27, and F29.

However, on functions F1, F4, F11, F14, F22, F25, and F30, MSHBA performs comparably to or slightly worse than the original Honey Badger Algorithm (HBA). On functions F10, F20, and F28, its performance is similar to that of HHO.

Overall, the improved Honey Badger Algorithm demonstrates enhanced optimization performance on the majority of the benchmark functions.

### 4.6. MSHBA for Test Function of Fitness Change Curve

The Multi-Strategy Honey Badger Algorithm (MSHBA) demonstrates superior convergence performance compared to the other five algorithms—COA, DBO, OOA, HHO, and HBA—on 29 benchmark functions, including F1 and F3–F19, F21–F30 (Figure 3, Figure 4, Figure 5, Figure 6, Figure 7, Figure 8, Figure 9, Figure 10, Figure 11, Figure 12, Figure 13, Figure 14, Figure 15, Figure 16, Figure 17, Figure 18, Figure 19, Figure 20, Figure 21, Figure 22, Figure 23, Figure 24, Figure 25, Figure 26, Figure 27, Figure 28, Figure 29, Figure 30 and Figure 31). Specifically, MSHBA outperforms COA, DBO, OOA, and HHO on functions F3, F20, and F29. Overall, the improved Honey Badger Algorithm exhibits significantly enhanced convergence behavior compared to the original HBA.

## 5. MSHBA for Solving Classical Engineering Problems

### 5.1. Weight Minimization of a Speed Reducer (WMSR) [51]

The reducer weight minimization problem is a typical engineering optimization problem, with the objective of achieving weight reduction in the reducer by optimizing design variables while satisfying a series of design constraints. The weight of the reducer depends on 11 constraints, which must be minimized. Among them, seven are nonlinear constraints, and the remaining four are linear constraints. The design variables include gear face width $b(x_{1})$, gear module $m(x_{2})$, number of teeth on the pinion $z(x_{3})$, bearing span of the first shaft $x_{4}$, bearing span of the second shaft $x_{5}$, diameter of the first shaft $d_{1}(x_{6})$, and diameter of the second shaft $d_{2}(x_{7})$. The mathematical model is established as follows:

Minimize

$$\begin{array}{l} F_{1}(\bar{x})=0.7854x_{2}^{2}x_{1}(14.9334x_{3}-43.0934+3.3333x_{3}^{2})\\ \;\;\;\;\;\;\;\;\;+0.7854(x_{5}x_{7}^{2}+x_{4}x_{6}^{2})-1.508x_{1}(x_{7}^{2}+x_{6}^{2})+7.477(x_{7}^{3}+x_{6}^{3}) \end{array}$$

subject to

$$\begin{array}{l} g_{1}(\bar{x})=-x_{1}x_{2}^{2}x_{3}+27\leq 0\\ g_{2}(\bar{x})=-x_{1}x_{2}^{2}x_{3}^{2}+397.5\leq 0\\ g_{3}(\bar{x})=-x_{2}x_{6}^{4}x_{3}x_{4}^{-3}+1.93\leq 0\\ g_{4}(\bar{x})=-x_{2}x_{7}^{4}x_{3}x_{5}^{-3}+1.93\leq 0\\ g_{5}(\bar{x})=10x_{6}^{-3}\sqrt{16.91\times 10^{6}+{\left(745x_{4}x_{2}^{-1}x_{3}^{-1}\right)}^{2}}-1100\leq 0\\ g_{6}(\bar{x})=10x_{7}^{-3}\sqrt{157.5\times 10^{6}+{\left(745x_{5}x_{2}^{-1}x_{3}^{-1}\right)}^{2}}-850\leq 0\\ g_{6}(\bar{x})=x_{2}x_{3}-40\leq 0,\;\;\;\;\;\;g_{8}(\bar{x})=-x_{1}x_{2}^{-1}+5\leq 0\\ g_{9}(\bar{x})=x_{1}x_{2}^{-1}-12\leq 0,\;\;\;g_{10}(\bar{x})=1.5x_{6}-x_{4}+1.9\leq 0\\ g_{11}(\bar{x})=1.1x_{7}-x_{5}+1.9\leq 0 \end{array}$$

with bounds

$$\begin{array}{l} 2.6\leq x_{1}\leq 3.6,\;\;\;0.7\leq x_{2}\leq 0.8,\;\;\;17\leq x_{3}\leq 28\\ 7.3\leq x_{4},x_{5}\leq 8.3,\;\;\;2.9\leq x_{6}\leq 3.9,\;\;\;5\leq x_{7}\leq 5.5 \end{array}$$

### 5.2. Tension/Compression Spring Design (TCSD) [52]

The design of tension/compression springs is a classic engineering optimization problem. The objective is to optimize the structural parameters of the spring to achieve minimum mass or optimal performance, while satisfying specific mechanical properties and spatial requirements. The design variables include wire diameter $d(x_{1})$, mean coil diameter $D(x_{2})$, and number of active coils $N(x_{3})$. The constraints are shear stress constraint, spring vibration frequency constraint, and minimum deflection constraint. The established mathematical model is as follows:

Minimize

$$F_{2}(\bar{x})=x_{1}^{2}x_{2}(2+x_{3})$$

subject to

$$\begin{array}{l} g_{1}(\bar{x})=1-\frac{x_{2}^{3}x_{3}}{71785x_{1}^{4}}\leq 0\;\\ \;\;g_{2}(\bar{x})=\frac{4x_{2}^{2}-x_{1}x_{2}}{12566(x_{2}x_{1}^{3}-x_{1}^{4})}+\frac{1}{5108x_{1}^{2}}-1\leq 0\\ g_{3}(\bar{x})=1-\frac{140.45x_{1}}{x_{2}^{2}x_{3}}\leq 0\\ g_{4}(\bar{x})=\frac{x_{1}+x_{2}}{1.5}-1\leq 0 \end{array}$$

with bounds

$$0.05\leq x_{1}\leq 2.00,0.25\leq x_{2}\leq 1.30,2.00\leq x_{3}\leq 15.0$$

### 5.3. Pressure Vessel Design (PVD) [52]

Pressure vessel design is a classic engineering optimization problem. The objective is to optimize the structural parameters of the pressure vessel to achieve cost minimization or performance optimization, while satisfying specific mechanical properties and safety standards. The design variables include shell thickness $T_{s}(x_{1})$, head thickness $T_{h}(x_{2})$, inner radius $R(x_{3})$, and shell length $L(x_{4})$. The constraints include thickness requirements, stress constraints, safety standard constraints, and geometric constraints. The established mathematical model is as follows:

Minimize

$$f(\bar{x})=1.7781z_{2}x_{3}^{2}+0.6224z_{1}x_{3}x_{4}+3.1661z_{1}^{2}x_{4}+19.84z_{1}^{2}x_{3}$$

subject to

$$\begin{array}{l} g_{1}(\bar{x})=0.00954x_{3}\leq z_{2}\\ \;\;g_{2}(\bar{x})=0.0193x_{3}\leq z_{1}\\ g_{3}(\bar{x})=x_{4}\leq 240\\ g_{4}(\bar{x})=-\pi x_{3}^{2}\leq -1296000 \end{array}$$

where

$$\begin{array}{l} z_{1}=0.0625x_{1}\\ z_{2}=0.0625x_{2} \end{array}$$

with bounds

$$\begin{array}{l} 1\leq x_{1},x_{2}\leq 99(\mathrm{int}eger\mathrm{var}iable)\\ 10\leq x_{3},x_{4}\leq 200 \end{array}$$

### 5.4. Welded Beam Design (WBD) [52]

Welded beam design is a classic engineering optimization problem. The objective is to optimize the structural parameters of the welded beam to achieve the minimization of manufacturing costs or the optimization of performance, while satisfying specific mechanical properties and safety standards. The design variables include weld thickness $h(x_{1})$, length of the beam attached to the support $l(x_{2})$, height of the beam $t(x_{3})$, and thickness of the beam $b(x_{4})$. The constraints include shear stress constraint, bending stress constraint, buckling load constraint, end deflection constraint of the beam, and geometric constraints. The established mathematical model is as follows:

Minimize

$$f(\bar{x})=0.04811x_{3}x_{4}(x_{2}+14)+1.10471x_{1}^{2}x_{2}$$

subject to

$$\begin{array}{l} g_{1}(\bar{x})=x_{1}-x_{4}\leq 0\\ g_{2}(\bar{x})=\delta (\bar{x})-\delta_{\max}\leq 0\\ g_{3}(\bar{x})=p\leq p_{c}(\bar{x})\\ g_{4}(\bar{x})=\tau_{\max}\geq \tau (\bar{x})\\ g_{5}(\bar{x})=\sigma (\bar{x})-\sigma_{\max}\leq 0 \end{array}$$

where

$$\begin{array}{l} \tau =\sqrt{{\tau^{\prime }}^{2}+{\tau^{\prime \prime }}^{2}+2\tau^{\prime }\tau^{\prime \prime }\frac{x_{2}}{2R}},\tau^{\prime \prime }=\frac{RM}{J},M=p(\frac{x_{2}}{2}+L)\\ R=\sqrt{\frac{x_{2}^{2}}{4}+{\left(\frac{x_{1}+x_{2}}{2}\right)}^{2}},J=2((\frac{x_{2}^{2}}{4}+{\left(\frac{x_{1}+x_{3}}{2}\right)}^{2})\sqrt{2}x_{1}x_{2})\\ \sigma (\bar{x})=\frac{6pL}{x_{4}x_{3}^{2}},\delta (\bar{x})=\frac{6pL^{3}}{Ex_{3}^{2}x_{4}},p_{c}(\bar{x})=\frac{4.013Ex_{3}x_{4}^{3}}{6L^{2}}(1-\frac{x_{3}}{2L}\sqrt{\frac{E}{4G}})\\ L=14in,p=6000lb,E=30.10^{6}psi,\sigma_{\max}=30000psi\\ \tau_{\max}=13600psi,G=12.10^{6}psi,\delta_{\max}=0.25in \end{array}$$

with bounds

$$\begin{array}{l} 0.1\leq x_{2},x_{3}\leq 10\\ 0.1\leq x_{4}\leq 2\\ 0.125\leq x_{1}\leq 2 \end{array}$$

**Figure 32:**
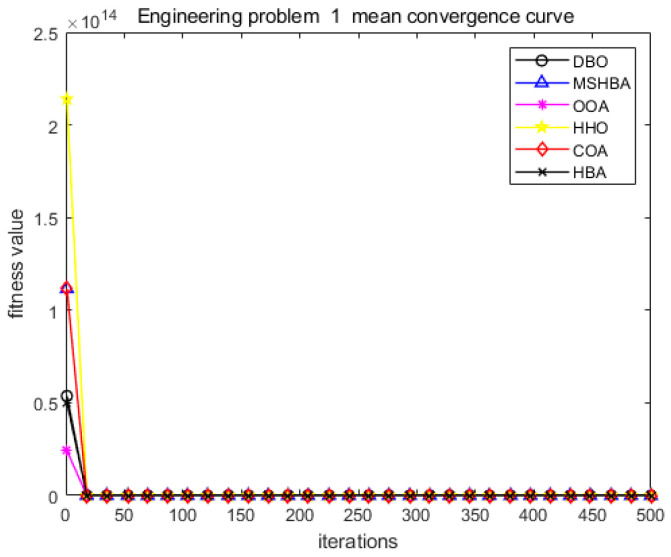
Convergence curve of problem 1.

**Figure 33:**
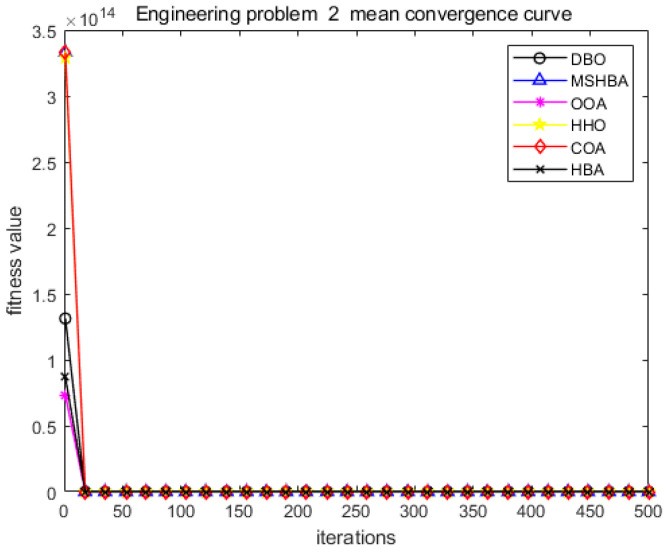
Convergence curve of problem 2.

**Figure 34:**
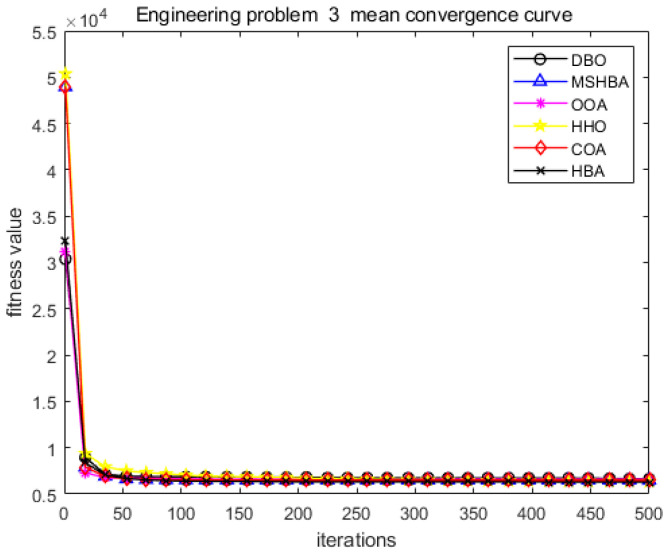
Convergence curve of problem 3.

**Figure 35:**
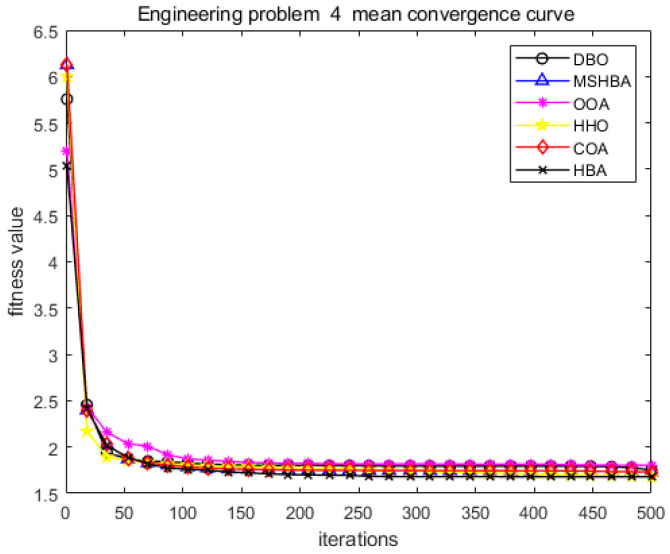
Convergence curve of problem 4.

The MSHBA algorithm demonstrates strong convergence performance and stability when solving constrained engineering design problems, as evidenced by the results presented in Table 8, Table 9, Table 10 and Table 11 and Figure 32, Figure 33, Figure 34 and Figure 35. Additionally, it can be observed from Table 12, Table 13, Table 14 and Table 15 that the decision variables achieve optimal solutions when the objective function reaches its optimum. The algorithm is capable of reaching the optimal objective function value during the initial iterations and demonstrates excellent stability. In summary, the improved Honey Badger Algorithm demonstrates outstanding performance in solving constrained engineering design problems.

## 6. Conclusions and Future Works

This paper proposes a hybrid strategy to enhance the performance of the Honey Badger Optimization Algorithm. Performance analysis further confirms the superior performance of MSHBA, as evidenced by its improved convergence rate and enhanced exploration–exploitation balance. However, the proposed Multi-Strategy Honey Badger Algorithm (MSHBA) employs a hybrid strategy to enhance the performance of the original Honey Badger Algorithm (HBA). Specifically, it introduces a cubic chaotic map during population initialization to improve the diversity and exploration capability of the initial population. In the excavation and honey-searching stages of the Honey Badger Algorithm, the position update mechanism relies on the global best solution, potentially resulting in premature convergence caused by population clustering around the optimal individuals. In order to improve the global exploration capability of the Honey Badger Algorithm, a random search strategy is incorporated to enhance convergence speed and computational efficiency in the later iterations.

In subsequent studies, the proposed MSHBA algorithm will be further assessed for its effectiveness in addressing multi-objective, combinatorial, and real-world optimization problems with complex and uncertain search spaces. Furthermore, integrating hybrid strategies and parameter adaptation techniques into the traditional Honey Badger Algorithm (HBA) can enhance its capabilities in binary and multi-objective optimization. This approach aims to improve solution accuracy, achieve a better balance between exploration and exploitation, and accelerate global convergence, thereby enabling the algorithm to effectively solve a wide range of optimization problems.

## Figures and Tables

**Figure 3:**
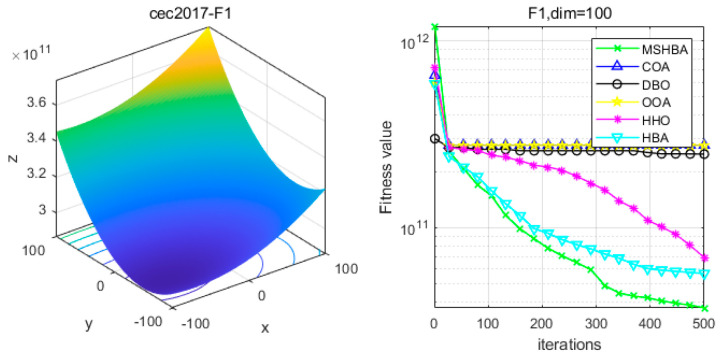
Convergence Performance Comparison of CEC2017-F1 Test Function.

**Figure 4:**
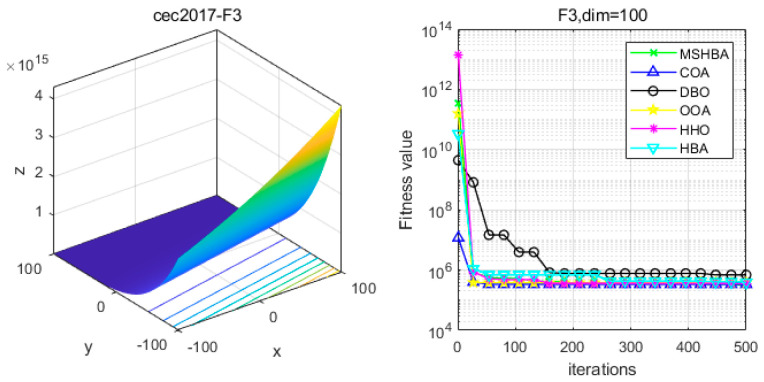
Convergence Performance Comparison of CEC2017-F3 Test Function.

**Figure 5:**
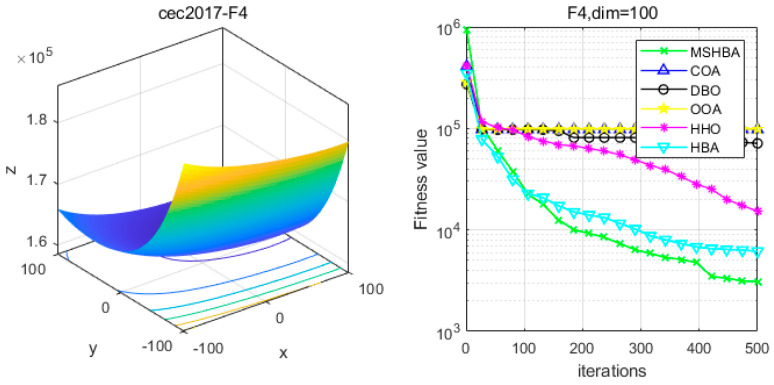
Convergence Performance Comparison of CEC2017-F4 Test Function.

**Figure 6:**
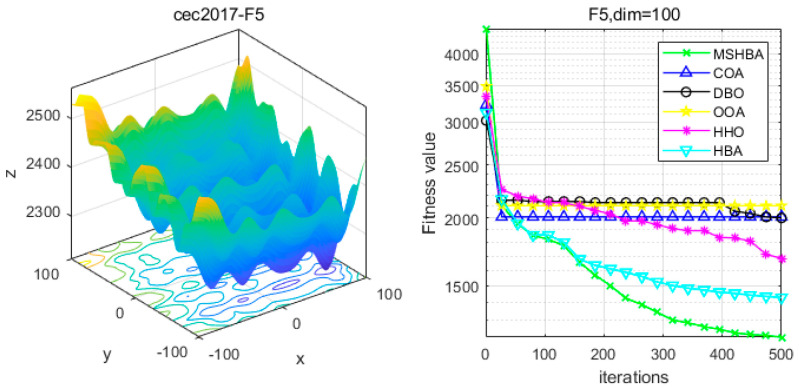
Convergence Performance Comparison of CEC2017-F5 Test Function.

**Figure 7:**
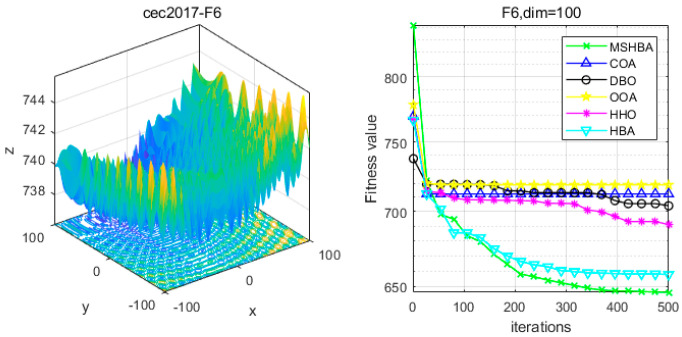
Convergence Performance Comparison of CEC2017-F6 Test Function.

**Figure 8:**
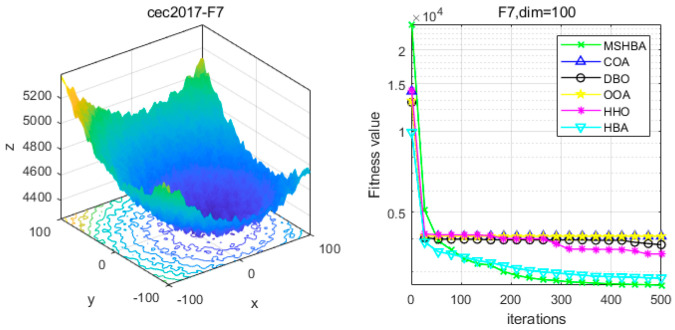
Convergence Performance Comparison of CEC2017-F7 Test Function.

**Figure 9:**
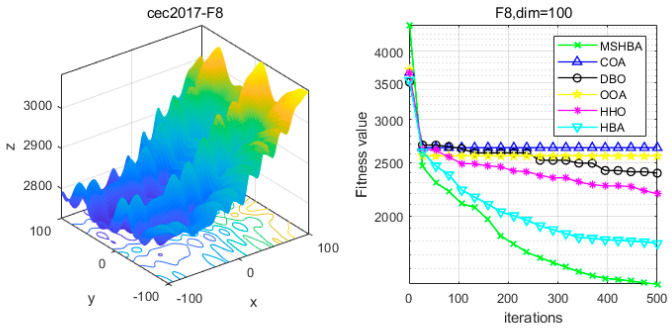
Convergence Performance Comparison of CEC2017-F8 Test Function.

**Figure 10:**
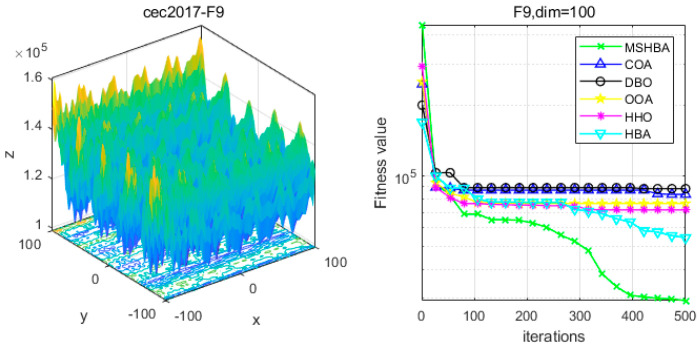
Convergence Performance Comparison of CEC2017-F9 Test Function.

**Figure 11:**
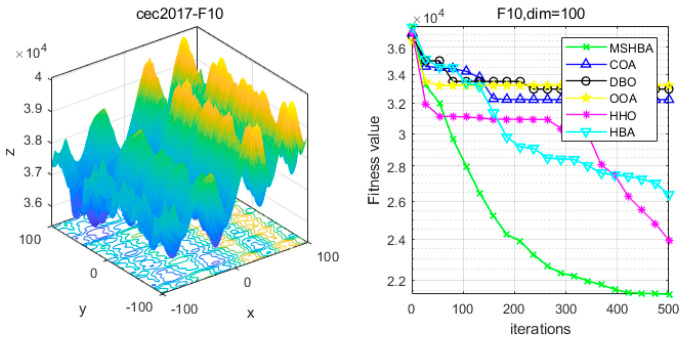
Convergence Performance Comparison of CEC2017-F10 Test Function.

**Figure 12:**
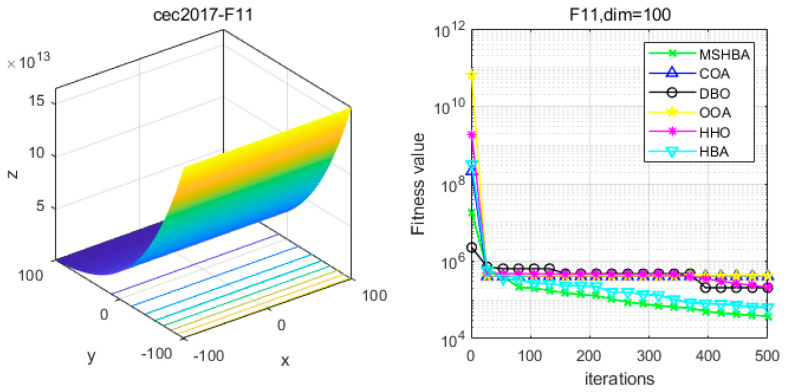
Convergence Performance Comparison of CEC2017-F11 Test Function.

**Figure 13:**
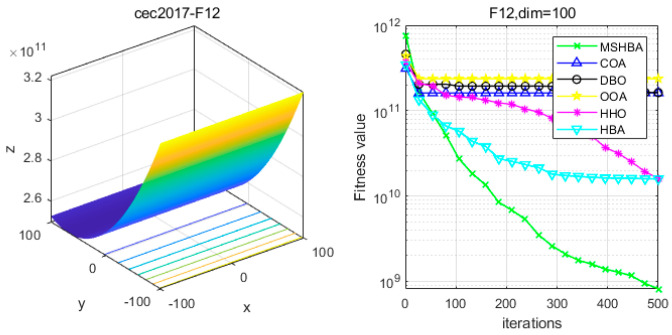
Convergence Performance Comparison of CEC2017-F12 Test Function.

**Figure 14:**
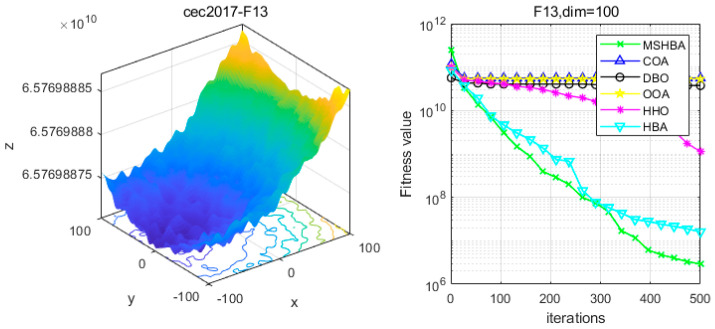
Convergence Performance Comparison of CEC2017-F13 Test Function.

**Figure 15:**
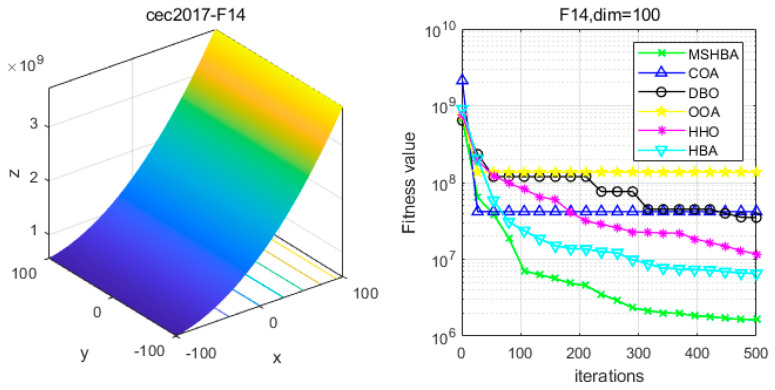
Convergence Performance Comparison of CEC2017-F14 Test Function.

**Figure 16:**
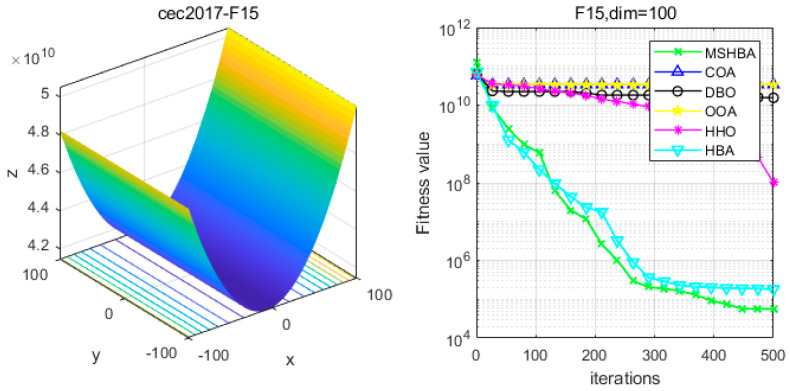
Convergence Performance Comparison of CEC2017-F15 Test Function.

**Figure 17:**
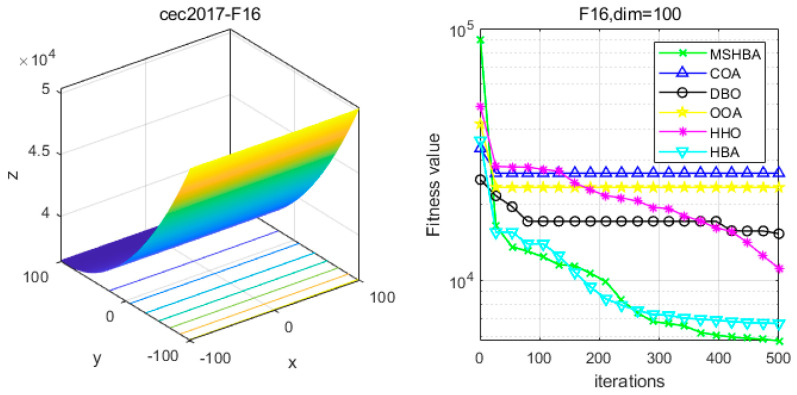
Convergence Performance Comparison of CEC2017-F16 Test Function.

**Figure 18:**
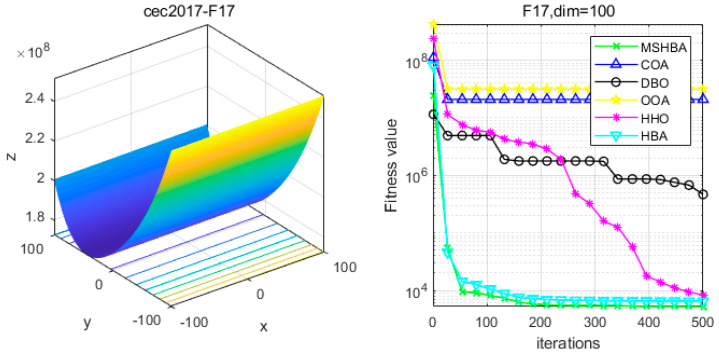
Convergence Performance Comparison of CEC2017-F17 Test Function.

**Figure 19:**
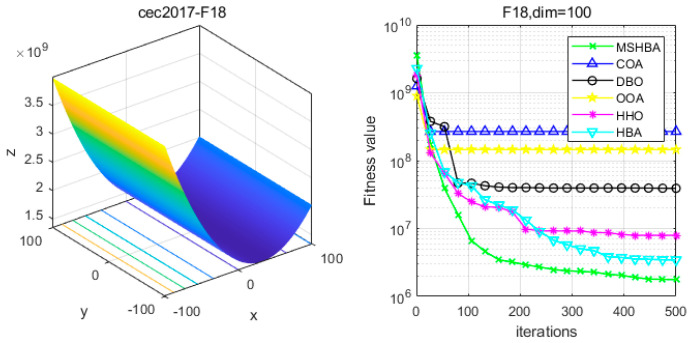
Convergence Performance Comparison of CEC2017-F18 Test Function.

**Figure 20:**
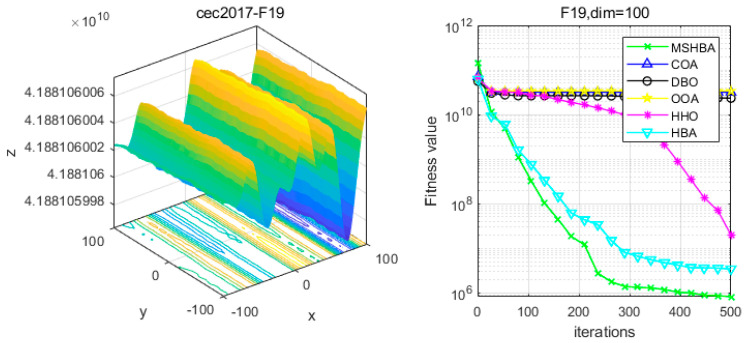
Convergence Performance Comparison of CEC2017-F19 Test Function.

**Figure 21:**
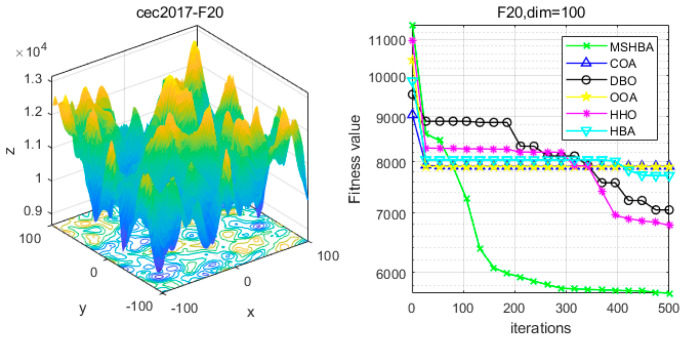
Convergence Performance Comparison of CEC2017-F20 Test Function.

**Figure 22:**
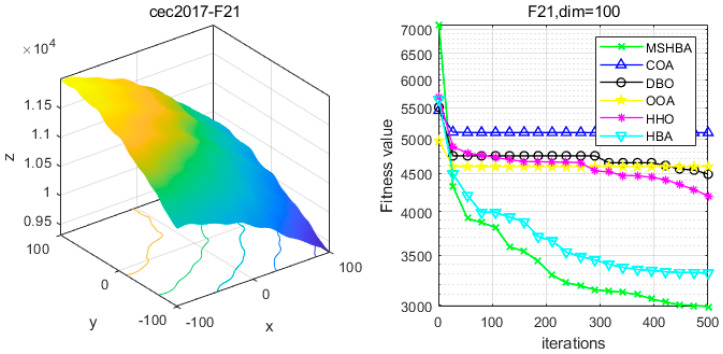
Convergence Performance Comparison of CEC2017-F21 Test Function.

**Figure 23:**
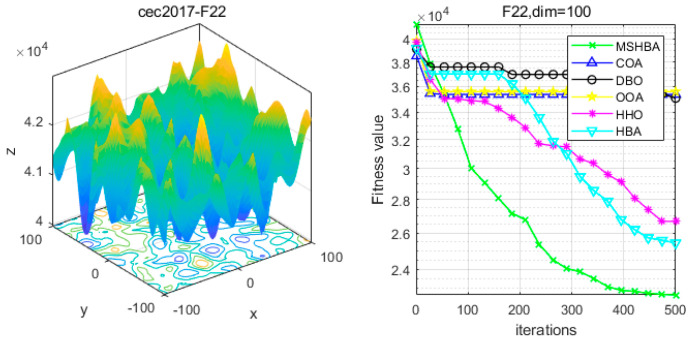
Convergence Performance Comparison of CEC2017-F22 Test Function.

**Figure 24:**
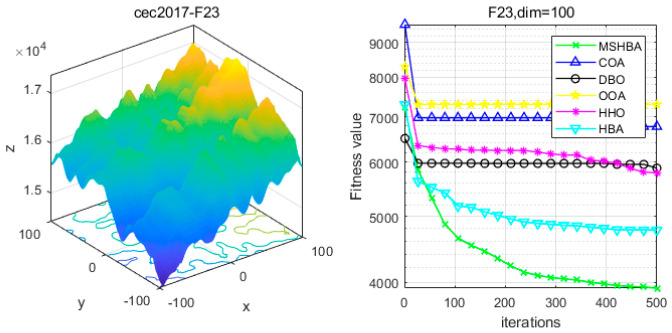
Convergence Performance Comparison of CEC2017-F23 Test Function.

**Figure 25:**
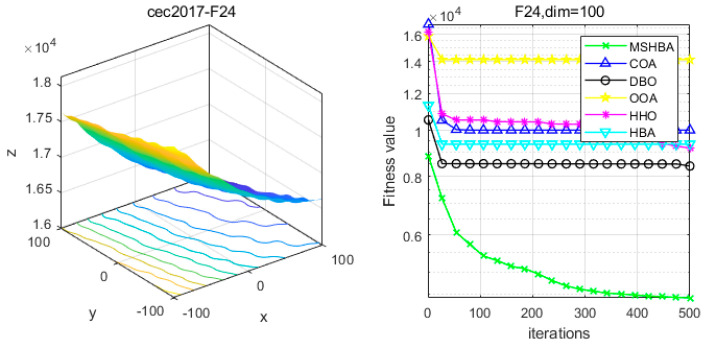
Convergence Performance Comparison of CEC2017-F24 Test Function.

**Figure 26:**
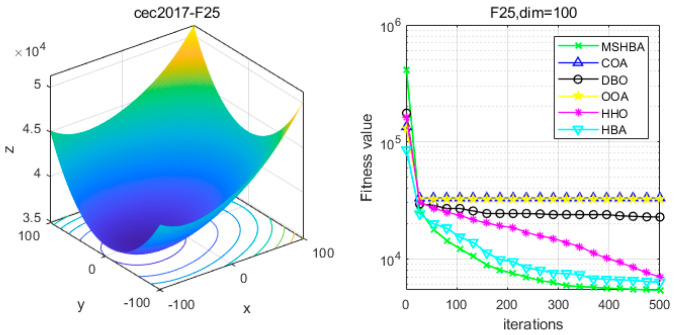
Convergence Performance Comparison of CEC2017-F25 Test Function.

**Figure 27:**
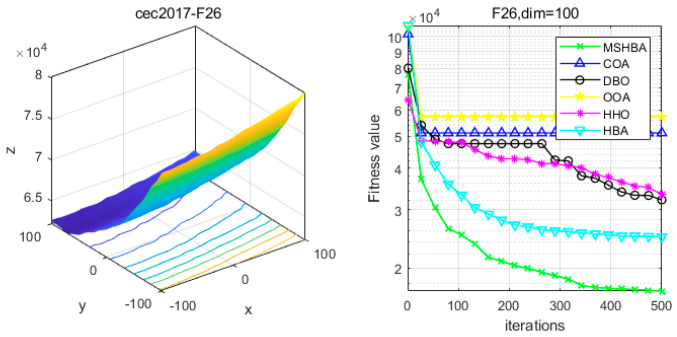
Convergence Performance Comparison of CEC2017-F26 Test Function.

**Figure 28:**
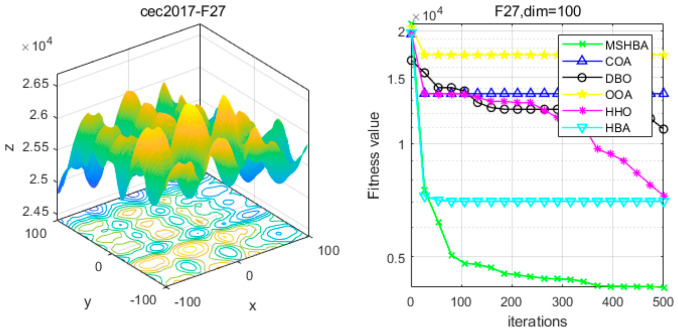
Convergence Performance Comparison of CEC2017-F27 Test Function.

**Figure 29:**
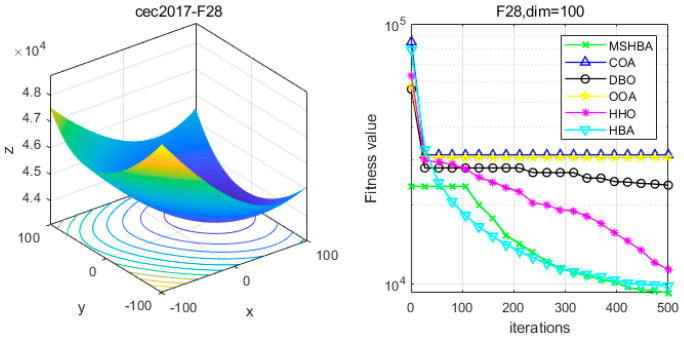
Convergence Performance Comparison of CEC2017-F28 Test Function.

**Figure 30:**
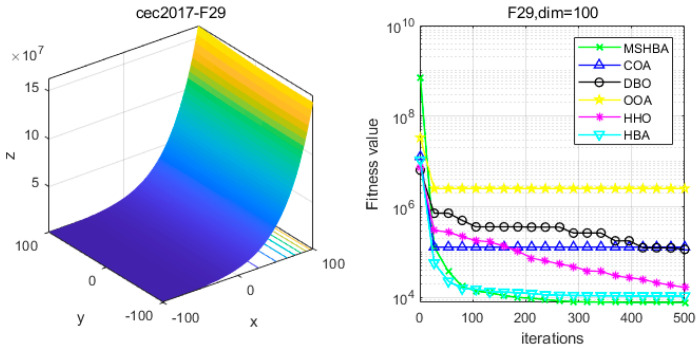
Convergence Performance Comparison of CEC2017-F29 Test Function.

**Figure 31:**
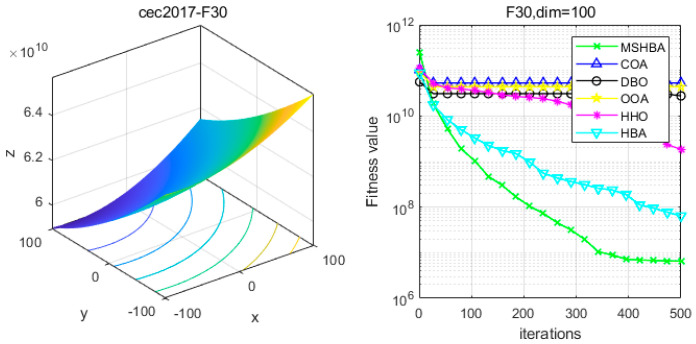
Convergence Performance Comparison of CEC2017-F30 Test Function.

**Table 1:** Summary of the CEC ’2017 test functions.

	No.	Functions	$F^{*}=F(x^{*})$
Unimodal Functions	1	Shifted and Rotated Bent Cigar Function	100
	3	Shifted and Rotated Zakharov Function	300
Simple Multimodal Functions	4	Shifted and Rotated Rosenbrock’s Function	400
	5	Shifted and Rotated Rastrigin’s Function	500
	6	Shifted and Rotated Expanded Scaffer’s F6 Function	600
	7	Shifted and Rotated Lunacek Bi_Rastrigin Function	700
	8	Shifted and Rotated Non-Continuous Rastrigin’s Function	800
	9	Shifted and Rotated Levy Function	900
	10	Shifted and Rotated Schwefel’s Function	1000
Hybrid Functions	11	Hybrid Function 1 (*N* = 3)	1100
	12	Hybrid Function 2 (*N* = 3)	1200
	13	Hybrid Function 3 (*N* = 3)	1300
	14	Hybrid Function 4 (*N* = 4)	1400
	15	Hybrid Function 5 (*N* = 4)	1500
	16	Hybrid Function 6 (*N* = 4)	1600
	17	Hybrid Function 6 (*N* = 5)	1700
	18	Hybrid Function 6 (*N* = 5)	1800
	19	Hybrid Function 6 (*N* = 5)	1900
	20	Hybrid Function 6 (*N* = 6)	2000
Composition Functions	21	Composition Function 1 (*N* = 3)	2100
	22	Composition Function 2 (*N* = 3)	2200
	23	Composition Function 3 (*N* = 4)	2300
	24	Composition Function 4 (*N* = 4)	2400
	25	Composition Function 5 (*N* = 5)	2500
	26	Composition Function 6 (*N* = 5)	2600
	27	Composition Function 7 (*N* = 6)	2700
	28	Composition Function 8 (*N* = 6)	2800
	29	Composition Function 9 (*N* = 3)	2900
	30	Composition Function 10 (*N* = 3)	3000
Search Range: [−100, 100]^*D*^			

**Table 2:** Parameters settings of IHBA and selected algorithm.

Algorithm	Parameters
COA	coati number = 30
	$t_{\max}$= 500
HHO	harris hawk number = 30
	$t_{\max}$= 500
DBO	dung beetle number = 30
	$t_{\max}$= 500
OOA	osprey number = 30
	$t_{\max}$= 500
HBA	honey badger number = 30
	$t_{\max}$= 500, β=6,C=2
MSHBA	honey badger number = 30
	$t_{\max}$= 500, β=6,C=2

**Table 3:** Unimodal functions.

Function	Index	MSHBA	HBA	COA	DBO	OOA	HHO
F1	min	36,332.21327	75,683.73185	44,680,368,018	24,217,484,929	42,002,907,856	100,012,066.4
F1	std	16,173,366.95	168,770,925.9	7,033,529,135	4,766,539,484	8,294,013,405	345,955,374.9
F1	avg	4,000,756.794	33,619,961.06	59,731,161,880	32,021,958,760	56,223,524,199	439,575,119.5
F1	median	601,797.5757	675,483.5145	59,521,568,792	31,651,854,438	55,292,058,030	359,305,702.1
F1	worse	89,307,263.31	925,953,472.7	70,695,016,541	45,351,521,182	69,887,934,634	1,665,291,319
F3	min	8168.289781	19,637.28281	79,071.67443	66,751.90092	73,028.09308	29,344.23558
F3	std	4174.231446	7984.245853	5087.478975	7426.879035	8097.010339	7765.054029
F3	avg	15,583.03135	39,438.34153	90,069.39714	83,047.78088	92,758.2175	56,224.97662
F3	median	15,452.63833	39,285.10263	91,008.4233	84,055.46423	92,718.38541	57,878.3185
F3	worse	25,626.58143	53,569.16705	102,566.6383	97,344.63217	106,866.2551	65,724.612

**Table 4:** Simple multimodal functions.

Function	Index	MSHBA	HBA	COA	DBO	OOA	HHO
F4	min	**471.2650759**	429.8653134	8676.540438	2280.188398	8831.397677	575.0612356
F4	std	**25.33961218**	43.35479888	3016.503386	2310.15102	2999.807664	92.66355947
F4	avg	**515.5658011**	520.6357708	15,449.90137	7926.726708	15,696.97266	696.2467487
F4	median	**515.2685906**	517.3036816	15,432.69729	7380.836144	16,168.57155	686.963974
F4	worse	**577.3558248**	655.374618	20,979.75537	13,948.7729	21,098.8796	988.4041393
F5	min	**539.0543882**	579.7459582	876.9745056	800.7163328	867.1474351	728.1264447
F5	std	**18.20793472**	29.64264405	25.76207594	25.19753215	29.87765232	31.15405432
F5	avg	**574.301356**	632.031401	924.9641367	847.6968692	932.0657073	771.1397853
F5	median	**572.9181152**	627.5478741	924.0502838	847.4039296	940.4132239	761.3208434
F5	worse	**604.1481834**	682.386896	973.0416522	898.4303847	973.4967331	838.8982555
F6	min	**601.0876771**	611.1461317	665.2970351	662.3393043	672.4335121	651.8707445
F6	std	**1.781596467**	8.878423374	7.281224277	5.15257125	7.909286248	6.135355419
F6	avg	**603.6710611**	624.3606534	689.514728	672.996196	686.6871553	668.0205421
F6	median	**603.3518742**	622.8009585	690.0552519	673.1831749	686.0670717	669.1685594
F6	worse	**609.0684128**	643.7839895	701.1380974	688.384032	700.7970491	678.3770071
F7	min	**768.6980104**	833.5550622	1293.006629	1152.28046	1315.449088	1094.740061
F7	std	**28.66807361**	52.49891689	52.54483794	49.95929834	51.01027465	70.4876899
F7	avg	**830.1325411**	918.1773428	1415.336924	1235.876865	1424.787752	1296.86546
F7	median	**825.2929227**	910.1463155	1423.410195	1241.328018	1431.421863	1299.322915
F7	worse	**890.7828372**	1077.243893	1490.873207	1366.684645	1521.818079	1446.083503
F8	min	**831.0104332**	858.9079183	1095.554037	999.9313048	1100.350225	893.4379959
F8	std	**19.90774063**	23.66975249	29.29692127	28.47589637	25.34583604	29.25608386
F8	avg	**870.4349257**	911.7121488	1149.864364	1068.882781	1142.133401	978.6668202
F8	median	**869.1223147**	914.0218159	1160.078501	1071.807161	1142.377033	981.8118432
F8	worse	**919.1643449**	944.7739496	1205.976809	1129.482433	1198.86356	1051.396815
F9	min	**959.6080177**	1423.433841	6152.58414	5931.118374	7591.509447	6594.499632
F9	std	**395.2917037**	1300.922409	1812.590238	1337.351272	1321.566109	1082.513028
F9	avg	**1378.269659**	3472.94881	10,986.97698	8787.753614	10,324.26437	8567.879885
F9	median	**1309.063502**	3491.843097	11,539.11699	8729.923302	10,502.01987	8495.984721
F9	worse	**2725.432474**	7361.937422	13,698.38918	11,540.95279	12,481.84783	11,199.1604
F10	min	**4458.587604**	3460.782787	7955.326892	5450.488897	7973.199151	4769.375371
F10	std	**1173.509975**	797.6259678	404.9918699	853.6256714	387.2710695	702.6015629
F10	avg	**6400.185464**	5481.735706	8748.598856	8428.448871	8846.051736	6105.540435
F10	median	**6220.904127**	5403.067257	8732.714625	8671.413401	8974.089012	6014.950217
F10	worse	**9396.640306**	6946.520619	9410.282349	9321.163997	9490.914007	7373.924473

**Table 5:** Hybrid functions.

Function	Index	MSHBA	HBA	COA	DBO	OOA	HHO
F11	min	**1151.199156**	1216.423405	6495.47519	3090.359845	5484.64063	1373.055653
F11	std	**57.83557438**	75.42573148	1587.626307	1310.326628	2394.807543	244.8888555
F11	avg	**1262.325404**	1330.453477	8809.916041	5676.5758	9412.856873	1623.867058
F11	median	**1260.735078**	1309.604659	8710.004238	5763.694408	9777.235861	1560.535589
F11	worse	**1423.229704**	1503.400433	11763.67689	8596.097835	13,794.18423	2627.061678
F12	min	**74,900.73363**	54,677.46578	8,004,724,178	789,307,016.7	8,182,408,482	13,090,571.92
F12	std	**1,345,797.802**	1,313,184.577	3,913,546,223	2,659,696,551	3,045,040,816	52,989,396.93
F12	avg	**1,099,451.974**	1,568,902.392	14,865,255,255	6,982,785,082	13,644,525,715	84,803,095.81
F12	median	**442,557.8368**	1,176,808.69	15,136,388,350	7,476,784,291	13,123,772,480	68,615,743.72
F12	worse	**5,792,037.187**	4,574,308.946	20,744,229,988	11,789,738,550	20,395,893,476	210,539,888.9
F13	min	**4539.3718**	6150.165678	1,489,333,852	397,260,539	1,817,805,403	286,183.994
F13	std	**25,110.96118**	824,912.7205	4,505,373,761	2,632,621,263	5,200,050,627	813,240.6509
F13	avg	**32,644.09**	208,033.3399	10,597,566,249	3,442,195,466	9,670,358,613	1,066,786.477
F13	median	**19,401.24954**	45,344.86841	10,172,352,017	3,644,524,954	8,417,691,801	836,432.0973
F13	worse	**72,977.0981**	4,566,519.743	24,141,559,320	10,745,888,750	20,809,917,388	4,074,357.755
F14	min	**1711.831641**	3326.949485	112,724.0795	38,819.47772	231,396.1531	47,760.74738
F14	std	**5936.700152**	32,243.53052	2,155,628.537	825,536.1505	9,825,484.425	885,781.4279
F14	avg	**6989.518107**	33,974.40606	2,191,192.564	798,810.2527	7,100,756.591	873,504.5218
F14	median	**4602.076785**	19,658.03254	1,616,476.92	627,218.5244	3,353,309.042	514,502.6535
F14	worse	**28,745.95484**	123,532.4077	11,557,981.02	3,576,983.465	45,832,920.4	3,472,437.752
F15	min	**2250.446281**	2959.719795	5,121,496.847	280,652.7269	55,165,095.95	24,165.02678
F15	std	**13,856.94895**	23,486.89054	471,974,341.2	36,434,849.5	561,614,606.5	49,771.67436
F15	avg	**13,675.28316**	18,354.87093	611,253,276.5	12,629,448.73	690,445,609.7	118,023.198
F15	median	**5277.728094**	11,497.20992	470,200,572.1	3,714,150.257	456,713,103.9	117,371.5719
F15	worse	**43,960.42823**	121,903.6222	1,550,944,089	203,183,415	2,071,388,517	253,191.8004
F16	min	**2012.847914**	2080.678415	4374.877498	3423.942514	4651.21137	3055.309339
F16	std	**216.1472784**	286.7054735	897.7786711	365.8196407	1070.803158	473.6407211
F16	avg	**2386.539097**	2605.07065	5770.466952	4112.25227	5881.963404	3731.242326
F16	median	**2421.91985**	2585.41721	5780.02071	4072.127174	5587.376156	3654.007111
F16	worse	**2859.637434**	3122.262691	7372.069495	4816.439998	8935.997457	5239.164841
F17	min	**1742.372139**	1852.49437	2180.186923	2443.362849	2652.781695	2163.695568
F17	std	**173.9290205**	224.6373734	2928.131984	270.8783634	5932.261031	297.7302024
F17	avg	**2070.236317**	2392.484763	4779.393382	2881.051447	6107.295908	2808.588621
F17	median	**2043.869238**	2408.685995	3782.066417	2864.486819	4961.477045	2848.983717
F17	worse	**2393.895527**	2833.384685	17,991.16568	3314.360198	34,438.94604	3373.572517
F18	min	**20,702.75367**	31,131.69771	3,827,698.722	753,574.3712	705,790.5116	126,809.6891
F18	std	**216,050.8949**	2,160,010.371	38,061,181.03	5,430,566.948	66,536,117.46	5,914,002.752
F18	avg	**229,876.8588**	729,865.1567	42,928,859.6	6,213,346.655	59,235,659.85	3,859,932.405
F18	median	**163,273.2064**	238,578.3376	31,545,216.57	5,021,293.509	38,632,192.56	1,369,412.165
F18	worse	**944,167.6911**	12,062,231.49	162,754,395.1	24,131,433.9	239,549,282.8	22,515,593.58
F19	min	**2101.48023**	2313.362816	15,734,537.68	24,800,002.02	16,199,347.56	106,105.1687
F19	std	**14,150.43313**	19,935.19608	535,984,424.3	146,049,396	400,151,587.5	1,256,830.774
F19	avg	**12,701.80058**	18,755.67073	550,228,302.6	188,822,826.3	583,411,721.3	1,485,477.19
F19	median	**6789.886063**	6281.857242	473,418,261.1	162,652,180.2	538,321,091.8	1,268,606.797
F19	worse	**54,393.0664**	56,697.17763	2,215,792,102	813,804,423	1,523,573,922	6,134,312.848
F20	min	**2184.836364**	2190.038131	2669.009695	2474.725265	2737.773396	2498.676162
F20	std	**322.9574545**	255.6539361	202.9663686	186.1973247	160.9241292	214.2404172
F20	avg	**2734.554772**	2629.882676	3035.771044	2818.567668	3022.743016	2840.438917
F20	median	**2734.954573**	2613.020621	3052.304652	2857.109462	3007.500388	2863.102686
F20	worse	**3375.434061**	3284.237114	3347.476599	3183.688842	3397.753491	3187.744902

**Table 6:** Composition functions.

Function	Index	MSHBA	HBA	COA	DBO	OOA	HHO
F21	min	**2328.374724**	2361.606655	2674.255547	2298.81606	2645.305901	2474.986844
F21	std	**21.54562477**	38.07026351	50.71681329	100.6468838	52.20327744	45.93516289
F21	avg	**2361.621356**	2416.934175	2756.923906	2567.787301	2724.265894	2571.424771
F21	median	**2357.666469**	2406.345148	2746.03419	2589.163152	2712.186617	2576.02751
F21	worse	**2414.891953**	2522.446549	2874.9941	2686.106601	2824.396175	2680.325844
F22	min	**2303.011291**	2303.803668	7394.126495	4952.563534	6418.441063	2476.604061
F22	std	**2744.984439**	2699.275571	931.1823853	896.2348088	942.4142636	1299.944318
F22	avg	**5774.571329**	4396.5146	9541.698929	6635.578235	9424.101049	7201.58516
F22	median	**6346.938172**	2320.466744	9879.163007	6547.412031	9661.900511	7380.529293
F22	worse	**10,872.12746**	10,131.19902	10,808.01632	9530.784605	10,457.53553	9091.500067
F23	min	**2692.701686**	2716.74975	3341.941897	3011.274542	3417.293907	2931.615137
F23	std	**28.03445356**	108.1414638	129.6919588	93.6055587	176.4835198	125.9967206
F23	avg	**2725.141462**	2838.0616	3582.434161	3151.875958	3711.998019	3224.611813
F23	median	**2720.602588**	2815.566227	3615.876905	3150.723736	3725.884802	3241.020759
F23	worse	**2799.636423**	3335.541587	3865.707013	3410.174895	4027.514463	3500.775722
F24	min	**2854.03441**	2898.570428	3426.104423	3221.32036	3653.231463	3239.149426
F24	std	**22.94711896**	182.4346651	168.4636008	96.51085244	238.7841852	143.9425178
F24	avg	**2895.846485**	3068.665229	3811.681441	3378.431225	4082.877526	3486.40142
F24	median	**2894.773175**	2992.307582	3803.227557	3388.283471	4042.760674	3511.12618
F24	worse	**2936.830332**	3561.44684	4170.948434	3584.009764	4569.061377	3869.309011
F25	min	**2888.0339**	2889.844867	4161.153332	3749.612239	4145.77482	2954.389479
F25	std	**21.77833403**	19.55326434	471.1370306	294.308607	546.961207	31.74155552
F25	avg	**2916.041587**	2920.008898	5155.440152	4240.862875	5036.006864	3011.140246
F25	median	**2913.782999**	2921.419063	5091.019266	4245.502821	4923.789288	3011.853877
F25	worse	**2977.465659**	2956.292902	6111.927067	4807.583137	6440.720825	3090.130785
F26	min	**3868.10695**	2822.411128	9865.2935	6691.216754	9515.474664	4212.221834
F26	std	**261.0555857**	1040.547875	864.1190973	788.4414258	1171.659009	1197.068098
F26	avg	**4412.029682**	5011.391515	11,468.69385	8328.356632	11,584.74908	8015.494868
F26	median	**4401.137387**	5037.256627	11,510.98905	8549.826893	11,562.82741	8317.378243
F26	worse	**4954.540489**	6919.703493	13,334.83768	9603.896656	14,288.20873	9897.642704
F27	min	**3203.596686**	3217.64719	3764.298723	3397.702045	3735.00574	3276.890401
F27	std	**375.1604378**	173.3180073	501.2971062	150.2258588	534.7853183	205.0072178
F27	avg	**3418.462613**	3342.389701	4493.857869	3669.40638	4841.336833	3535.701627
F27	median	**3304.911169**	3307.459554	4424.195815	3657.460642	4843.963482	3487.872732
F27	worse	**4809.336071**	4068.668299	5725.68992	3956.127687	6093.658352	4273.873259
F28	min	**3207.537647**	3215.539111	6219.829655	4759.858161	6276.802658	3347.569615
F28	std	**964.1069312**	35.93018009	596.6879353	380.8594573	614.0424277	68.96732179
F28	avg	**3619.889274**	3282.614929	7422.944436	5542.163689	7385.34041	3474.906644
F28	median	**3273.387211**	3286.34706	7541.352154	5660.78289	7489.362639	3461.763521
F28	worse	**6912.590173**	3382.611969	8724.415482	6185.494938	8414.745851	3666.251144
F29	min	**3494.322658**	3467.102238	5827.179913	4377.03191	5186.816056	4153.204096
F29	std	**390.6260893**	287.9024078	1235.80597	360.3593788	1843.805616	395.5045007
F29	avg	**4024.048157**	4096.387997	7398.869217	5018.876752	8150.940429	5056.387627
F29	median	**3975.567817**	4111.554342	7068.020938	4970.159496	7617.972569	5160.819057
F29	worse	**4891.942631**	4820.404623	11,120.62393	5660.56642	12,418.38164	5796.888337
F30	min	**6596.26567**	7878.446768	568,099,420.9	22,276,646.83	262,230,934.4	2,198,313.919
F30	std	**6851.453476**	88,005.28982	903,137,928.5	289,290,425.3	1,130,305,003	9,307,191.494
F30	avg	**17,546.12975**	72,406.14781	1,713,122,036	214,934,810.1	1,920,168,273	11,292,240.97
F30	median	**16,024.00032**	30,456.11583	1,407,603,022	143,764,437.1	1,586,940,284	10,002,707.62
F30	worse	**42,228.44695**	386,843.3297	4,408,681,625	1,646,995,834	4,855,238,130	41,377,423.53

**Table 7:** Rank-sum test.

Function	HBA	COA	DBO	OOA	HHO
F1	**0.662734758**	3.01986 × 10^−11^	3.01986 × 10^−11^	3.01986 × 10^−11^	3.01986 × 10^−11^
F3	**4.97517 × 10^−11^**	3.01986 × 10^−11^	3.01986 × 10^−11^	3.01986 × 10^−11^	3.01986 × 10^−11^
F4	**0.589451169**	3.01986 × 10^−11^	3.01986 × 10^−11^	3.01986 × 10^−11^	3.68973 × 10^−11^
F5	**1.07018 × 10^−9^**	3.01986 × 10^−11^	3.01986 × 10^−11^	3.01986 × 10^−11^	3.01986 × 10^−11^
F6	**3.01986 × 10^−11^**	3.01986 × 10^−11^	3.01986 × 10^−11^	3.01986 × 10^−11^	3.01986 × 10^−11^
F7	**1.85673 × 10^−9^**	3.01986 × 10^−11^	3.01986 × 10^−11^	3.01986 × 10^−11^	3.01986 × 10^−11^
F8	**9.83289 × 10^−8^**	3.01986 × 10^−11^	3.01986 × 10^−11^	3.01986 × 10^−11^	4.97517 × 10^−11^
F9	**3.15889 × 10^−10^**	3.01986 × 10^−11^	3.01986 × 10^−11^	3.01986 × 10^−11^	3.01986 × 10^−11^
F10	**0.001679756**	8.48477 × 10^−9^	2.19589 × 10^−7^	5.96731 × 10^−9^	0.539510317
F11	**0.000268057**	3.01986 × 10^−11^	3.01986 × 10^−11^	3.01986 × 10^−11^	4.97517 × 10^−11^
F12	**0.072445596**	3.01986 × 10^−11^	3.01986 × 10^−11^	3.01986 × 10^−11^	3.01986 × 10^−11^
F13	**0.027086318**	3.01986 × 10^−11^	3.01986 × 10^−11^	3.01986 × 10^−11^	3.01986 × 10^−11^
F14	**9.06321 × 10^−8^**	3.01986 × 10^−11^	3.01986 × 10^−11^	3.01986 × 10^−11^	3.01986 × 10^−11^
F15	**0.129670225**	3.01986 × 10^−11^	3.01986 × 10^−11^	3.01986 × 10^−11^	1.09367 × 10^−10^
F16	**0.003033948**	3.01986 × 10^−11^	3.01986 × 10^−11^	3.01986 × 10^−11^	3.01986 × 10^−11^
F17	**1.02773 × 10^−6^**	7.38908 × 10^−11^	3.01986 × 10^−11^	3.01986 × 10^−11^	1.46431 × 10^−10^
F18	**0.065671258**	3.01986 × 10^−11^	4.07716 × 10^−11^	3.68973 × 10^−11^	3.19674 × 10^−9^
F19	**0.239849991**	3.01986 × 10^−11^	3.01986 × 10^−11^	3.01986 × 10^−11^	3.01986 × 10^−11^
F20	**0.180899533**	0.000253058	0.162375022	0.00033679	0.122352926
F21	**7.77255 × 10^−9^**	3.01986 × 10^−11^	1.54652 × 10^−9^	3.01986 × 10^−11^	3.01986 × 10^−11^
F22	**0.185766856**	5.0922 × 10^−8^	0.529782491	1.35943 × 10^−7^	0.023243447
F23	**1.41098 × 10^−9^**	3.01986 × 10^−11^	3.01986 × 10^−11^	3.01986 × 10^−11^	3.01986 × 10^−11^
F24	**1.41098 × 10^−9^**	3.01986 × 10^−11^	3.01986 × 10^−11^	3.01986 × 10^−11^	3.01986 × 10^−11^
F25	**0.340288465**	3.01986 × 10^−11^	3.01986 × 10^−11^	3.01986 × 10^−11^	4.97517 × 10^−11^
F26	**0.000268057**	3.01986 × 10^−11^	3.01986 × 10^−11^	3.01986 × 10^−11^	4.19968 × 10^−10^
F27	**0.641423523**	1.69472 × 10^−9^	7.69496 × 10^−8^	4.19968 × 10^−10^	4.63897 × 10^−5^
F28	**0.8766349**	8.99341 × 10^−11^	2.60151 × 10^−8^	8.99341 × 10^−11^	1.72903 × 10^−6^
F29	**0.206205487**	3.01986 × 10^−11^	9.7555 × 10^−10^	3.01986 × 10^−11^	1.41098 × 10^−9^
F30	**2.95898 × 10^−5^**	3.01986 × 10^−11^	3.01986 × 10^−11^	3.01986 × 10^−11^	3.01986 × 10^−11^

**Table 8:** Statistical results of competitor algorithms for WMSR problem.

Engineering 1	Index	MSHBA	COA	DBO	OOA	HHO	HBA
F1	min	2994.424466	2994.424466	2994.424467	3005.476722	2994.424466	2994.424466
F1	std	2.84848056	65.16978689	45.09751196	5.028371006	2.368455165	2.54734021
F1	avg	2995.358009	3056.1574	3034.296393	3012.829612	2995.046823	2995.376420
F1	median	2994.424466	3038.368266	3033.701596	3011.346302	2994.424466	2994.424466
F1	worse	3003.75982	3188.264544	3188.264544	3025.967663	3003.75982	3002.57892

**Table 9:** Statistical results of competitor algorithms for TCSD problem.

Engineering 2	Index	MSHBA	COA	DBO	OOA	HHO	HBA
F2	min	0.01266534	0.012676941	0.012669641	0.012669581	0.012667402	0.01266421
F2	std	0.00127634	0.00217847	0.000982581	0.000156243	0.001532633	0.001254356
F2	avg	0.013211802	0.014403461	0.013158595	0.012834463	0.013415017	0.013321041
F2	median	0.012719054	0.01312391	0.012843473	0.012763832	0.012719054	0.012718236
F2	worse	0.017773158	0.018026697	0.017773158	0.013347791	0.017773158	0.017764532

**Table 10:** Statistical results of competitor algorithms for PVD problem.

Engineering 3	Index	MSHBA	COA	DBO	OOA	HHO	HBA
F3	min	6059.714335	6059.71435	6059.714552	6060.37146	6059.714335	6061.823546
F3	std	512.5757289	579.9510833	316.2129992	374.7160367	540.7771321	524.34210589
F3	avg	6513.097363	6594.239536	6451.562474	6238.452261	6688.493798	6534.0238912
F3	median	6370.779717	6338.958449	6410.086778	6065.4648	6728.854785	6380.2384513
F3	worse	7544.492518	7544.492518	7332.843509	7425.013265	7544.492518	7678.5642312

**Table 11:** Statistical results of competitor algorithms for WBD problem.

Engineering 3	Index	MSHBA	COA	DBO	OOA	HHO	HBA
F3	min	1.670217919	1.672242773	1.670218795	1.6717035	1.670218056	1.670217919
F3	std	0.142683524	0.113967612	0.084676517	0.002774216	0.239656288	0.142683524
F3	avg	1.787526474	1.789799218	1.71024892	1.67635062	1.81837122	1.787526474
F3	median	1.72404532	1.771026028	1.670359559	1.676323843	1.724064807	1.72404532
F3	worse	2.183640071	2.282913448	1.979411719	1.682554819	2.679760746	2.183640071

**Table 12:** Statistical competition algorithm for solving WMSR problem.

Algorithm	X1	X2	X3	X4	X5	X6	X7
MSHBA	3.5	0.7	17	7.3	7.71532	3.35054	5.28665
COA	3.5	0.7	17	7.3	7.71532	3.35054	5.28665
DBO	3.5	0.7	17	7.3	7.71532	3.35054	5.28665
OOA	3.5	0.7	17	7.37735	7.93909	3.35091	5.28803
HHO	3.5	0.7	17	7.3	7.71532	3.35054	5.28665
HBA	3.5	0.7	17	7.3	7.71532	3.35054	5.28665

**Table 13:** Statistical competition algorithm for solving TCSD problem.

Algorithm	X1	X2	X3
MSHBA	0.05	0.317425	14.0278
COA	0.0526386	0.379993	10.0444
DBO	0.0514621	0.351283	11.6148
OOA	0.0505182	0.329028	13.1295
HHO	0.0519519	0.363074	10.9258
HBA	0.05	0.317425	14.0278

**Table 14:** Statistical competition algorithm for solving PVD problem.

Algorithm	X1	X2	X3	X4
MSHBA	12.84797	6.88227	42.09382	176.89271
COA	12.58581	7.04886	41.63912	182.41281
DBO	13.20307	6.57646	40.66877	195.19762
OOA	15.07952	7.73834	48.57513	110.06771
HHO	14.88946	8.10283	48.57513	110.06775
HBA	13.43681	6.95521	42.08866	177.10523

**Table 15:** Statistical competition algorithm for solving WBD problem.

Algorithm	X1	X2	X3	X4
MSHBA	0.19883	3.33741	9.19213	0.19883
COA	0.19795	3.36312	9.19134	0.19891
DBO	0.19775	3.35981	9.19012	0.19914
OOA	0.19563	3.48621	9.20923	0.19947
HHO	0.19932	3.44251	9.15293	0.20123
HBA	0.19673	3.37823	9.19134	0.19883

## Data Availability

Data is contained within the article: Theoriginal contributions presented in this study are included in the article. Further inquiries can be directed to the corresponding author.
